# DNMT3A and TET2 in the Pre-Leukemic Phase of Hematopoietic Disorders

**DOI:** 10.3389/fonc.2016.00187

**Published:** 2016-08-22

**Authors:** Hanae Sato, Justin C. Wheat, Ulrich Steidl, Keisuke Ito

**Affiliations:** ^1^Ruth L. and David S. Gottesman Institute for Stem Cell and Regenerative Medicine Research, Albert Einstein College of Medicine, Bronx, NY, USA; ^2^Department of Cell Biology, Albert Einstein College of Medicine, Bronx, NY, USA; ^3^Department of Anatomy and Structural Biology, Albert Einstein College of Medicine, Bronx, NY, USA; ^4^Albert Einstein Cancer Center, Albert Einstein College of Medicine, Bronx, NY, USA; ^5^Department of Medicine, Montefiore Medical Center, Albert Einstein College of Medicine, Bronx, NY, USA; ^6^Einstein Diabetes Research Center, Albert Einstein College of Medicine, Bronx, NY, USA

**Keywords:** TET2, Dnmt3a, myelodysplastic syndromes, acute myeloid leukemia, pre-LSC, stem cell biology, epigenetic regulator, HSCs

## Abstract

In recent years, advances in next-generation sequencing (NGS) technology have provided the opportunity to detect putative genetic drivers of disease, particularly cancers, with very high sensitivity. This knowledge has substantially improved our understanding of tumor pathogenesis. In hematological malignancies such as acute myeloid leukemia and myelodysplastic syndromes, pioneering work combining multi-parameter flow cytometry and targeted resequencing in leukemia have clearly shown that different classes of mutations appear to be acquired in particular sequences along the hematopoietic differentiation hierarchy. Moreover, as these mutations can be found in “normal” cells recovered during remission and can be detected at relapse, there is strong evidence for the existence of “pre-leukemic” stem cells (pre-LSC). These cells, while phenotypically normal by flow cytometry, morphology, and functional studies, are speculated to be molecularly poised to transform owing to a limited number of predisposing mutations. Identifying these “pre-leukemic” mutations and how they propagate a pre-malignant state has important implications for understanding the etiology of these disorders and for the development of novel therapeutics. NGS studies have found a substantial enrichment for mutations in epigenetic/chromatin remodeling regulators in pre-LSC, and elegant genetic models have confirmed that these mutations can predispose to a variety of hematological malignancies. In this review, we will discuss the current understanding of pre-leukemic biology in myeloid malignancies, and how mutations in two key epigenetic regulators, *DNMT3A* and *TET2*, may contribute to disease pathogenesis.

## Introduction

One striking finding from NGS studies has been the wide range in the number of mutations that appear in different tumor types; while some tumors can contain thousands of changes in coding sequences, such as bladder adenocarcinoma and melanoma, other tumors have a paucity of genetic aberrations (1, 2). Hematological malignancies, such as acute myeloid leukemia (AML) and myelodysplastic syndromes (MDS), generally fall within this latter category, where it seems that only a handful of exome mutations are necessary and sufficient to drive these malignancies (1–5), although these studies cannot rule out that mutations in regulatory elements and non-coding regions are the putative causal hits in these diseases. Nevertheless, while the low frequency of coding mutations in hematological malignancies would suggest that they would be simpler to manage clinically and more amenable to targeted therapies, clinical trials using selective inhibitors of supposed driver mutations in the bulk tumor population have been largely disappointing thus far and have not been able to achieve lasting remission (6–14). Intensive chemotherapy regimens with stem cell transplantation remain the standard of care (15, 16). Additionally, while clinical remission is achieved in a substantial proportion of AML cases, most patients will relapse and die from their disease. Determining why tumors with such low mutational burden are refractory to targeted therapy and why these patients relapse so frequently are therefore critical questions in the field.

Hematopoiesis is a complex and plastic differentiation system that requires the temporally coordinated expression of large cohorts of genes in progressively more differentiated hematopoietic stem and progenitor cells [HSPC; reviewed by Dick and Lapidot; Doulatov et al. (17, 18)]. These fate decisions have empirically been proven to result from the stoichiometry between master transcription factors (19–22). Global regulators of transcription, such as epigenetic and chromatin remodeling complexes, splicing factors, and the core transcription machineries, also play an important upstream role in establishing the correct transcriptional landscape to allow for the efficient activation or repression of target genes. Interestingly, a number of NGS studies have identified a growing list of mutations within these factors in MDS and AML (Table 1). Both MDS and AML are not single diseases, but rather collections of clinically related but phenotypically heterogeneous malignancies. MDS are classically thought of as a disease of hematopoietic stem cells (HSC), in which patients have either marked reductions in blood production, at times precipitating into bone marrow failure, significant dysplasia in the cells produced, or some combination of both. AML are at least eight separate morphological phenotypes [French–American–British (FAB) classification system] that involve a large expansion of immature blast populations. The clinical hallmark of these malignancies is a differentiation block and thus substantial defects in the generation of mature erythrocytes, platelets, and/or mononuclear cells such as lymphocytes, neutrophils, and monocytes. In both AML and MDS, there appears to be multiple parallel clones and subclones that undergo evolutionary competition during disease progression, producing a clonal hierarchy within each patient (5, 23–27). Importantly, these clones may have different susceptibilities to treatment options and therefore represent important reservoirs during disease relapse (28–30). Additionally, these diseases are not stagnant but continue to evolve over time; this is best exemplified by the finding that some patients with MDS will convert to AML over the course of their treatment.

**Table 1:** **Examples of somatic mutations identified in AML and MDS**.

	AML	MDS	Protein names
Signal transduction (Class I)	*FLT3*	*FLT3*	Fms-related tyrosine kinase 3
	*c-KIT*		KIT proto-oncogene receptor tyrosine kinase
	*N-Ras, K-Ras*	*N-Ras, K-Ras*	Neuroblastoma and Kirsten Rat Sarcoma Viral (V-Ras) Oncogene Homolog
	*JAK2*	*JAK2*	Janus Kinase 2
		*CBL*	Casitas B-lineage lymphoma
Transcription (Class II)	*CEBP* α		CCAAT/enhancer-binding Protein alpha
	*IKZF1*		IKAROS family zinc finger 1
	*RUNX1*	*RUNX1*	Runt-related transcription factor 1 (or AML1)
	*PHF6*		PHD finger protein 6
Epigenetic regulation	*TET2*	*TET2*	Ten eleven translocation methylcytosine Dioxygenase 2
	*IDH1/2*	*IDH1/2*	Isocitrate dehydrogenase-1 and -2
	*DNMT3A*	*DNMT3A*	DNA methyltransferase 3A
	*ASXL1*	*ASXL1*	Additional sex combs like transcriptional regulator 1
	*EZH2*	*EZH2*	Enhancer of Zeste Homolog 2
RNA splicing	*U2AF1*		U2 small nuclear RNA auxiliary factor 1
	*SF3B1*	*SF3B1*	Splicing factor 3b, subunit 1
	*SRSF2*	*SRSF2*	Serine/arginine-rich splicing factor 2
		*ZRSR2*	Zinc finger (CCCH Type), RNA-binding motif, and serine-/arginine-rich 2
Tumor suppressor	*CDKN2A/B*		Cyclin-dependent kinase inhibitor 2A
	*TP53*	*TP53*	Tumor Protein p53
	*WT1*	*WT1*	Wilms Tumor 1
Other	*SMC1A*		Structural maintenance of chromosomes 1A
	*NPM1*	*NPM1*	Nucleophosmin

One remarkable finding of AML and MDS has been the discovery of mutations that produce very different clinical phenotypes even when these mutations occur in the same cell. For instance, a mutation in some gene *X* in HSC could produce bone marrow failure as typified by conditions like aplastic anemia or MDS, or could produce a blastic like disease of more differentiated progenitor compartments as in AML. Furthermore, mutations occurring in the bulk tumor population can also frequently be found within supposedly “normal” HSPC that are contributing to multi-lineage differentiation (26, 28, 31). These findings have suggested the existence of a theorized pre-leukemic stem cells (pre-LSC). These pre-LSC are fundamentally distinct from the tumor initiating, CD34^+^ CD38^−^ leukemia stem cells (LSC or leukemia-initiating cells, LIC) described extensively over the past two decades (32–34). Pre-LSC, are clones within the hematopoietic hierarchy that are not proliferative or dysplatic, but are inherently more likely to transform into a frank leukemia at a higher rate than other HSPC clones. These pre-LSC contain a limited number of mutations in AML or MDS related genes, such as *TET2, DNMT3A*, or *ASXL1*, and have qualitative changes that make them leukemogenic, this is in stark contrast to non-pre-LSC clones in patients with clonal hematopoiesis of indeterminate potential (CHIP; see Discussion and Perspectives below). Importantly, pre-LSC are thought to contribute to normal hematopoiesis while slowly accumulating mutations until a critical number are reached to produce LSC. These LSC, having crossed some threshold, then give rise to MDS or AML while not contributing to normal hematopoiesis. Determining the identity of the genes required to cross this “leukemic threshold,” knowing within which cell they arise, and knowing what order they occurred are the critical questions in the field.

## Evidence for Pre-LSC in Human Myeloid Malignancies

**Figure 1:**
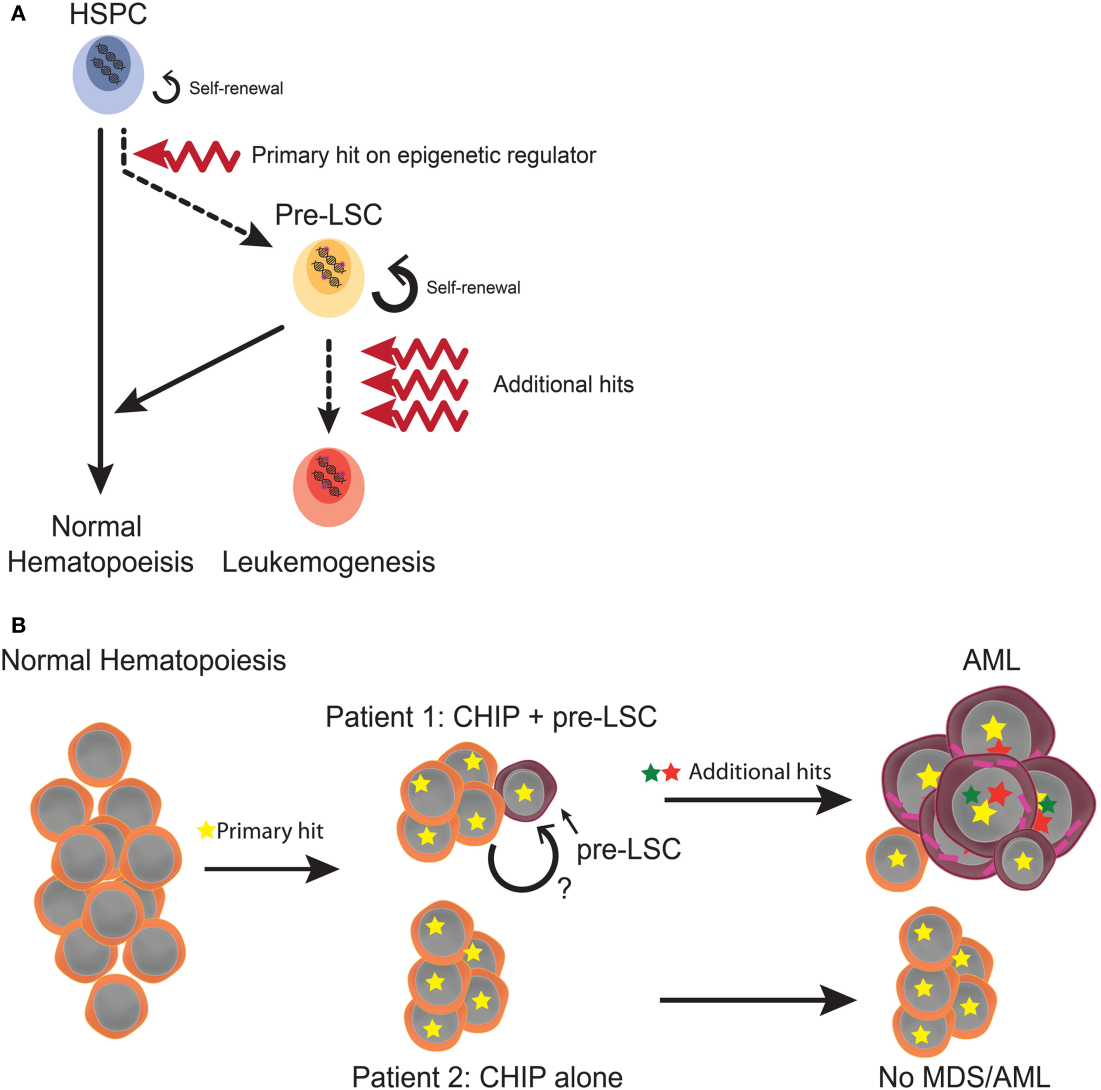
Hypothetical model of leukemogenesis in *TET2* and *DNMT3A* mutations **(A)** Model of the stepwise mutation accumulation during pre-leukemic hematopoiesis and leukemogenesis. Numerous studies have suggested that mutations converting HSPC to pre-leukemic stem cell (pre-LSC) are in epigenetic regulators and may lead to aberrant transcriptional networks utilized in both HSC self-renewal and differentiation. When additional hits are then acquired in these pre-LSC, leukemia develops. Importantly, pre-LSC still contribute to normal hematopoiesis and self-renew similar to normal HSC (indicated in both by solid arrows) until malignant transformation (indicated with broken red arrow) in the pre-LSC model. **(B)** Hypothetical model of CHIP and pre-LSC. CHIP is defined as oligoclonal hematopoiesis in the presence of an AML or MDS mutation yet without cytopenia or dysplasia. Pre-LSC are phenotypically normal clones harboring mutations in AML and MDS genes, and can occur in CHIP or in non-CHIP patients. The major theoretical difference between pre-LSC and HSC participating in CHIP is the propensity to transform once additional hits are obtained. While these hits do not readily transform other CHIP clones, pre-LSC clones can rapidly progress to fully malignant state. The qualities that confer this “primed leukemic state” are currently unknown but presumably account for why not all patients with CHIP develop AML or MDS, as indicated in the figure. HSPC, hematopoietic stem and progenitor cell; CHIP, clonal hematopoiesis of indeterminate potential; AML, acute myeloid leukemia; MDS, myelodysplastic syndromes.

The possibility of pre-leukemic HSC harboring leukemia-associated mutations is not novel. In 1987, Fialkow and colleagues first identified presumably leukemic clones contributing to normal erythropoiesis in acute non-lymphocytic leukemia (35). Building on seminal work by Lapidot and colleagues, which first established that CD34^+^ CD38^−^, but not blasts, from AML patients, constituted LSC and could initiate leukemia in xenotransplanted mice (36). Hope et al. (37) further demonstrated that these xenotransplanted LSC could undergo further clonal evolution in these mice, and later studies showed that these cells could even produce normal myeloid and lymphoid lineages (38). Recapitulating these findings in xenotransplantation models, Miyamoto et al. found that *AML1/ETO* transcripts, which are generated due to the leukemic translocation *t*(8:21) in AML, are detectable in mature blood cells of all lineages even after stable and complete remission (39). Then, in 2012, Jan et al. reported a pioneering study in the pre-LSC field using flow cytometry and single clone targeted re-sequencing (26). By following the frequency and co-occurrence of many mutations within the same patient longitudinally during therapy and at disease relapse, these authors constructed the most detailed maps to date describing clonal dynamics in human AML. Moreover, by isolating residual “normal” HSC from these patients (defined as CD99^−^ TIM3^−^ CD34^+^ CD38^−^ Lin^−^), they identified a number of mutations in critical epigenetic regulators frequently mutated in AML and MDS. The authors proposed a model whereby the disease propagating LSC are derived initially from these residual, self-renewing HSPCs that harbored primary mutation(s) (Figure 1A). These mutations presumably maintained these pre-LSC in a “primed” state that was able to expand into AML LSC once a driving mutation was acquired. Importantly however, these residual pre-LSC but not putative LSC (CD99^+^ CD34^+^ CD38^−^ Lin^−^ cells) were contributing to normal, multi-lineage hematopoiesis, again deviating significantly from prior cancer stem cell (CSC) models, whereby the CSC was incapable of generating normal tissue. Studies previously showing that LSC were capable of multilineage reconstitution were presumably assaying these same pre-LSC as those studies only used CD34 and CD38 to enrich for LSC (38).

In addition to providing the best evidence to date for pre-LSC, this work and studies by a number of other groups have since made the remarkable discovery that the mutations occurring in pre-LSC and the bulk tumor were categorically different: while early mutations in pre-LSC were frequently in epigenetic and chromatin remodeling regulators, driver mutations in myeloid transcription factors and signal transduction molecules such as tyrosine kinases tended to occur late in bulk blast cells (40, 41). This surprising finding not only helped explain why potent targeted therapies for some driver mutations failed to cure patients; it also suggested something fundamental about AML biology and the order in which mutations were acquired.

Based on these observations, a number of groups started investigating how mutation sequence affects clinical outcomes in myeloid malignancies. If leukemia did indeed arise from pre-LSC harboring mutations that primed cells for leukemogenesis, then one would predict that the order within which mutations were acquired might influence the clinical phenotype. Ortmann et al. (42) tested this hypothesis by determining the mutational order between the epigenetic regulator *Ten-Eleven Translocation 2* (*TET2*) and a putative driver *JAK2 V617F*. These genes have been reported to be mutated in both pre-LSC and fully transformed malignant disorders such as the Philadelphia chromosome negative myeloproliferative neoplasms (MPN) [such as primary myelofibrosis (PMF), essential thrombocythemia (ET), and polycythemia vera (PV)], AML, and MDS. In this study, the authors focused on the MPNs, ET and PV, where the same *JAK2* mutations are almost universal in both conditions despite very different clinical phenotypes, and sought to determine whether the order of *TET2* and *JAK2* mutations along the hematopoietic lineage and within malignant clones drove differences in the clinical phenotype of the MPN. They found that the mutation order of *TET2* and *JAK2 V617F* influenced the age when the MPN was diagnosed, the subclonal composition and proliferative capacity of flow cytometry defined HSPC in these patients, and the transcriptional profile of the malignant HSC (42). Similar observations were additionally reported for *DNA Methyltransferase 3 alpha* (*DNMT3A*) mutations in MPN (43). However, the most interesting finding from these studies was that the clinical manifestations of disease were also significantly influenced by mutational order: acquiring either *TET2* or *DNMT3A* mutation prior to the *JAK2* mutation resulted in a much higher frequency of ET rather than PV.

In recent years, a number of important sequencing studies have also established that while hematopoietic clonality can influence clinical outcomes, identifying clones with certain mutations carries much more prognostic information. Two whole-exome sequencing studies of peripheral blood mononuclear cells longitudinally tracked clonal hematopoiesis during aging to establish whether the presence of clonal hematopoiesis correlated with AML development (5, 27). In both studies, the authors found that clonal hematopoiesis became more common with older age, that patients with clonal hematopoiesis had slightly higher rates of AML, and that the most common mutations in these clones were in *ASXL1, TET2*, and *DNMT3A*. Consistent with these results, Yoshizato et al. found strong evidence in aplastic anemia patients that while the degree of clonal hematopoiesis was variable and itself not well correlated with overall survival, clones harboring mutations in the epigenetic modifiers *ASXL1* and *DNMT3A* expanded much more rapidly than other clones in the same patient and that patients carrying these types of clones had significantly poorer overall survival and higher rates of transformation to AML (44). Recently, another group described the presence of chemotherapy-resistant HSC in patients with AML after chemotherapy that appeared to expand rapidly upon depletion of the bulk tumor (28). Again, these clones harbored mutations in epigenetic modifiers commonly seen in AML patients and therefore may represent the expansion of leukemia primed pre-LSC in these patients. Finally, Ivey and colleagues found that minimal residual disease, which was detected by the presence of *Nucleophosmin* (*NPM1*)-mutated transcripts in normal mononuclear cells after achieving remission in AML, was one of the strongest predictors of relapse and carried a poor overall survival. While this study was not addressing pre-LSC mutations *per se*, the fact that finding oncogenic transcripts in normal mature blood cells after AML remission carried a strongly poor prognosis clearly fits well with a model of pre-LSC being primed to transform rapidly into relapse AML despite contributing to normal hematopoiesis (45). Taken together, it appears that pre-LSC have qualitative changes that make them distinctly more prone to leukemia initiation than other HSC.

## Discriminating CHIP from Pre-Leukemia

Although the evidence for pre-LSC has garnered substantial support owing to the work described above, one question has plagued both clinical and translational studies: why do some patients with clonal hematopoiesis harboring mutations in MDS and AML-associated genes never develop disease? In the studies by Jaiswal et al. and Genovese et al. mentioned above, the majority of patients with clonal hematopoiesis harboring mutations in canonical preleukemic mutations (e.g., *TET2, ASXL1*, or *DNMT3A*) never developed MDS or AML. This realization has complicated our understanding of pre-LSC as it suggests that preleukemic mutations are not fully sufficient to generate the “pre-LSC state” than primes for leukemic transformation. To help describe this scenario whereby a patient has limited hematopoietic clonality and harbors preleukemic mutations but does not have an increased risk of AML or MDS, a number of translational researchers and clinicians have described a clinical entity called “clonal hematopoiesis of indeterminate potential,” or CHIP, which is analogous to monoclonal gammopathy of unknown significance (MGUS). CHIP is defined as oligoclonal hematopoiesis without morphological changes or cytopenia, where one or more genes typically associated with AML or MDS are mutated. CHIP patients have a very low rate of conversion to AML or MDS and therefore (and unlike in MDS) can be monitored clinically rather than proactively treated. A more detailed description of CHIP, the research leading to its characterization, and the diagnostic criteria separating it from MDS and AML are discussed extensively by Steensma and colleagues (46) and are beyond the scope of this review. While CHIP is clearly different from frank MDS or AML, discriminating CHIP from pre-leukemia is nuanced. CHIP is a risk classifier that describes, clinically, the probability of a patient to develop leukemia. Pre-LSC are cells that deterministically drive AML or MDS. While CHIP patients absolutely have an increased risk for these malignancies, the overall risk is still quite low. Pre-LSC, on the other hand, are fundamentally primed to contribute to leukemia initiation: according to the current model of leukemogenesis, all AML and MDS patients have pre-LSC that contribute to normal hematopoiesis, the bulk leukemia, and relapse. Therefore, all CHIP patients that develop AML had a resident pre-LSC clone in their CHIP and it does appear that CHIP seems to increase the risk of developing a pre-LSC. However, not all patients with CHIP will ever develop a pre-LSC and therefore will never develop MDS or AML. Moreover, not all AML patients originally had CHIP (Figure 1B). Importantly, the qualitative distinction that makes pre-LSC leukemogenic in patients with or without CHIP does not need to be genetic: epigenetic differences, metabolic rates, cell extrinsic influences, or the transcriptional context of that particular HSC may discriminate what is a primed pre-LSC from a normal HSC that happens to harbor an AML associated mutation. As such, exome capture alone is unlikely to fully capture why these clones are inherently more likely to transform. Unfortunately, as cell surface markers have not been discovered that unambiguously capture only pre-LSC from other HSC or CHIP clones, this model cannot be tested empirically at this time (for a more detailed description of the differences between HSC, pre-LSC, CHIP, and LSC, along with relevant references, see Table 2). Furthermore, we cannot exclude the possibility that all clones in CHIP would, if provided enough time, become pre-LSC and generate leukemia; it is completely possible (and indeed highly plausible) that this is an entirely stochastic process of trait acquisition. Nevertheless, irrespective of the difficulties in separating these clinical subtleties with current technology, the evidence outlined above clearly shows that pre-LSC are real biological entities. Moreover it is also certainly true that the mutations isolated from pre-LSC must have a role in transforming pre-leukemic hematopoiesis to AML and MDS, as the presence of these mutations absolutely increases the probability of leukemogenesis. As noted, studies have repeatedly shown that many of these candidate antecedent mutations in pre-LSC are in epigenetic regulators. Given these observations, a substantial amount of effort has been directed at understanding how mutations in epigenetic factors deregulate hematopoiesis and precipitate hematological malignancies.

**Table 2:** **Cell types that associate with leukemoginesis and their cell surface markers**.

Cell type	Hematopoietic lineage potential	Leukemogenic	Presence of AML/MDS mutations?	Cell surface markers
HSC	Yes	No	No	Lin^-^CD34^+^CD38^-^CD90^+^ (39)
	Multilineage contribution to all mature blood populations, self-renewal			
Progenitors	Yes	No	No	Many, e.g., GMP: Lin^-^CD34^+^CD38^+^CD45RA^+^CD123^+^ (47)
	Restricted differentiation potential			
LSC	No	Yes	Yes	Lin^-^CD34^+^CD38^-^. Many reported markers, CLL-1 (48), CD25 (49), CD32 (49), CD96 (50), TIM-3 (51, 52), CD99 (52), CD47 (53), IL3RA (54)
pre-LSC	Yes	Yes	Yes	Unclear. Reports suggest Lin^-^CD34^+^CD38^-^TIM3^-^CD99^−^ (52) or Lin^-^CD34^+^CD38^-^IL1RAP^+^ (55)
	Multilineage contribution to all mature blood populations, self-renewal			
				No definitive marker available
CHIP	Yes	Minimal risk	Yes	Unclear. Presumably same as HSC
	Multilineage contribution to all mature blood populations, self-renewal			

*GMP, granulocyte-macrophage progenitor; IL3RA, interleukin 3 receptor; TIM-3, T-cell immunoglobulin and mucin domain 3; IL1RAP, IL-1 receptor accessory protein*.

## Mutations in DNA Methylation Regulators

**Figure 2:**
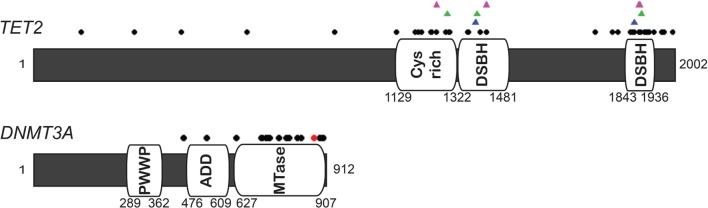
**Diagram of human *TET2* and *DNMT3A* and missense mutations in AML and MDS**. The black dots indicate the missense mutation sites (66–71). The majority of mutations in *TET2* are identified in the two catalytic domains, shown as double-stranded β-helix (DSBH) and Cys-rich domain (64, 72). Magenda, green, and blue triangles on *TET2* diagram represent the site associated with *N*-oxalylglycine (NOG, a 2-OG analog), CpG recognition and Fe(II) binding based on the crystal structure and biochemical analysis (73, 74). Red dot on *DNMT3A* indicates the hot spot mutation at R882.

Since epigenetic modifications regulate genome wide transcriptional profiles and help establish cell-type specific gene expression profiles during cell differentiation (56), mutations in these genes in HSPC may have profound effects on normal hematopoiesis. Two of the best-characterized epigenetic mutations found in pre-leukemic HSPC are in *DNMT3A* and *TET2* (Figure 2). *DNMT3A* and *DNMT3B* are *de novo* methyltransferases that catalyze DNA methylation at target DNA, while *DNMT1* is responsible for maintenance methylation at the replication folk during DNA synthesis (57–59). *De novo* DNMTs are essential to mammalian development (60, 61) as these marks, particularly 5-methylcytosine (5-mC) in CpG islands, are correlated with transcriptional silencing. *TET2*, conversely, is an enzyme that plays a central role in DNA demethylation by catalyzing the conversion of 5-mC to 5-hydroxymethyl cytosine (5-hmC) (62–64). While first discovered in 1972 (65), the functional importance of 5-hmC was not clear until recently due to the high mutational frequency of *TET2* in myeloid malignancies (Table 1). We will now focus on mutations in these enzymes in MDS and AML to shed insight into the role these factors play in hematopoiesis and leukemia.

## Regulation of *DNMT3A* and Its Role in Transcriptional Control

*DNMT3A* is a *de novo* methyltransferase of DNA and mutations have been isolated in patients with AML (69, 71) and MDS (70, 75). *DNMT3A* mutations frequently co-occur with *NPM1, FLT3*, and *IDH1* (76), and overall approximately 20% of patients with AML (69, 71) and 8% of patients with MDS (70) carry a mutation in this factor. Patients harboring mutations in *DNMT3A* typically have a poorer overall prognosis, although this depends significantly on which cooperating mutations co-occur in that patient (69–71).

The importance of DNA methylation in determining cell identity is well documented, but how *de novo* methyltransferase activity is regulated and how methyltransferases are targeted to specific DNA sites remains poorly understood. Recent studies have suggested strong cross-talk between histone modifications, transcriptional activity, and prior DNA methylation status on DNMT localization. Highly conserved PWWP (proline–tryptophan–tryptophan–proline) domains in *DNMT3A* play an essential role in directing this factor to heterochromatic regions (77, 78), particularly those marked with histone 3 lysine 36 tri-methylation (H3K36me3), which is a known repressive histone modification. Presence of this mark was also reported to increase the methyltransferase activity of DNMT3A (79). Protein–protein interactions with other factors also appear to play an important role in DNMT3A recruitment. *DNMT3A* has an ADD [ATRX-DNMT3- DNMT3-like (DNMT3L)] domain, which is a cysteine-rich (Cys-rich) zinc-finger DNA-binding domain, reported to interact with many transcription factors and chromatin remodeling factors such as HP1, SUV39H1 (80), EZH2 (81), HDAC1 (82), p53 (83), Myc (84), and PU.1 (85) (Figure 2). This domain in *DNMT3A* also shows high affinity for unmethylated histone H3 peptides but not H3 lysine 4 tri-methylated (H3K4me3) peptides (86–88), indicating that the ADD may also be involved in chromatin reading as well. Interestingly, based on the crystal structure of DNMT3A and after biochemical analyses, it was found that the ADD participates in an auto-regulatory capacity to effect changes in DNMT3A activity: in the absence of histone H3, ADD domain binds to the catalytic domain of DNMT3A leading to blocked enzymatic function; in the presence of unmethylated H3, the ADD binds this H3, allowing the catalytic domain to become accessible for *de novo* DNA methylation catalysis (89).

A final, important regulatory step controlling DNMT3A activity is tetramer formation. DNMT3A can exist in a variety of tetramer states composed of homo-dimers of DNMT3A or hetero-dimers with DNMT3L, which is a catalytically inactive protein that enhances the methyltransferase activity of DNMT3A (90). While all tetramers are catalytically active, each variant tetramer has marked differences in enzyme processivity (91). Therefore, understanding the regulation of tetramer formation, and specifically determining the mechanisms underlying homo- versus hetero-dimerization may be critical to understanding the regulation of DNMT3A function. Recent studies have also noted that these tetramers are sensitive to pH and decreasing pH disrupted the distribution of various tetramers of DNMT3A *in vitro* (91). As different cell types, and notably cancer cells, exist at slight variations in pH, destabilization of DNMT3A tetramers due to changes in intracellular pH may represent a relatively unexplored mechanism by which DNA methylation patterns are deregulated in tumors (92, 93).

## *DNMT3A* Mutations in AML and MDS

About 40–60% of *DNMT3A* mutations in AML patients are a hotspot mutation in Arg882 (R882), which is located within the catalytic domain of the enzyme (69, 71) (Figure 2). In addition to presumably reducing the catalytic efficiency of DNMT3A, this hotspot mutation also appears to influence the ability of DNMT3A to homodimerize. Normally, DNMT3A functions as a tetramer, comprised of either two homodimers or heterodimers DNMT3L. While R882 mutations in *DNMT3A* are still able to undergo hetero-dimerization with DNMT3L (94), they are unable to homo-dimerize (95, 96), suggesting that the R882 mutation is a dominant-negative mutation, which interrupts tetramer formation leading to the reduction of methyltransferase activity. *DNMT3A* mutations probably were inducing AML by leading to passive demethylation of the genome, and some genes (e.g., *HOXB*) have been found to be differentially hypomethylated in *DNMT3A* mutant AML (71). One recent study in murine HSC has indicated that many genes deregulated in leukemia, including transcription factors, exist in sites termed methylation canyons that are prone to methylation loss in the absence of *DNMT3A* (97). These results, however, conflict with whole genome profiling using Methylated DNA IP (MeDIP)-chip analysis and gene expression profiling that have thus far found little impact of *DNMT3A* mutations on global methylation patterns and little correlation between changes in methylation status and differential gene expression (69). One possible interpretation of this finding is that *DNMT3A* mutations play a more important role in pre-LSC transcriptional changes in HSPC that allow for leukemia to develop in more differentiated blasts, and that these changes are in effect “averaged-out” with standard ensemble techniques. Consistent with this idea is the finding that in inducible mouse deletion models of *Dnmt3a*, HSPC have mild phenotypic changes such as impaired differentiation, increased self-renewal, and occasionally transform to a myeloproliferative disease, but do not show robust changes in DNA methylation patterns or correlation between methylation changes and gene expression profiles (98–101). Double knockouts for *Dnmt3a* and *Dnmt3b*, however, show synergism in their phenotype, suggesting that there may exist compensatory activity between the *de novo* DNMTs in murine HSC that reduces the impact of single gene loss (98, 101). Moreover, inducible overexpression of *Dnmt3b* in mice was able to significantly slow leukemia induction by both *Myc-Bcl* and *Mixed Lineage Leukemia* (*MLL*)*-AF9* (102). These findings in mice raise an important question as to why *DNMT3B* mutations are so rare in human AML and MDS, indicating that perhaps this compensatory pathway is not as robust in human HSC (76).

## Functional Role of TET2 in Transcriptional Regulation

While 5-hmC-modified DNA was biochemically isolated decades ago, it was the recent discovery of *TET* mutations in AML and MDS that prompted further investigation of the functional role in these marks, and their writers the *TET* enzymes, play in transcriptional regulation. *TET1* was the first *TET* family member successfully isolated, originally found as a translocation partner of *MLL* gene in AML (103–105). While Ono and colleagues were the first to clone the gene and named it *LCX* (*leukemia-associated protein with a CXXC domain*) (106); Lorsbach et al. (107) cloned the same partner of the *MLL* translocation and named it *TET* for *Ten-Eleven Translocation* owing to its frequent *MLL* fusion [*t*(10;11)(q22;q23)] in AML. Three TET enzymes, TET1, 2, and 3, have since been identified (107).

All three TET enzymes convert 5-mC to 5-hmC, which is later converted to 5-formylcytosine (5-fC) and then 5-carboxylcytosine (5-caC) (62–64). While each enzyme is capable of catalyzing these reactions, expression profiling has shown cell type distribution differences between the different TET enzymes, indicating distinct functions or regulators (62). Classically, conversion of 5-mC to 5-hmC at promoters and transcription start sites (TSS) would be predicted to lead to transcriptional activation by eliminating DNA methylation, which is correlated with transcriptional repression at CpG islands. Williams et al. (108), however, reported an unexpected role for TET1 as a transcription repressor in embryonic stem cells. Moreover, other groups have found that TET1, but not TET2, interacts with the transcriptional repressive histone deacetylase SIN3A (108, 109). While TET2 is still typically believed to be involved in transcriptional activation, these non-canonical activities of other TET family members at least leaves open the possibility that TET2 may have as of yet unidentified regulatory roles in transcription. One recent finding is that TET2 can regulate histone *O*-acetylglucosaminylation (*O*-GlcNAcylation) of serine and threonine residues of histone 2B (H2B), which is reported to associate with active transcription at TSS (110). Chen et al. (111) found that TET2 regulates these levels indirectly by recruiting *via* its catalytic C terminus *O*-GlcNAc transferases (OGT) to target loci. Importantly, this interaction does not affect the 5-hmC catalytic activity of TET2 (111–113).

## Regulators of TET Activity

While different cell types seem to express different amounts of each TET enzyme, it has become clear that post-translational regulation is critical in controlling TET activity and targeting to genetic loci. All TET enzymes contain one Cys-rich domain and two double-stranded β-helix (DSBH) domains that display the core catalytic domains, which act in a Fe(II) and 2-oxoglutarate (2-OG, also called as α-ketoglutarate) dioxygenases-dependent manner (73). Mono ubiquitinylation at a conserved lysine residue (residue 1299 in TET2) (114, 115) or binding of ascorbic acid in this catalytic domain directly facilitates TET catalytic activity by stabilizing Fe(II) association with the enzyme (116, 117) (Figure 2).

Targeting of *TET2* to genomic regions was initially unclear as *TET2*, unlike *TET1* or *TET3*, does not possess a canonical CXXC domain that binds unmethylated CpG. Ko et al. (118) then found an ancestral variant of the CXXC domain, referred to as *IDAX* (a.k.a. *CXXC4*) 650 kb upstream of *TET2*, which appears to have been separated from the *TET2* coding region by chromosome inversion during evolution. IDAX interacts with unmethylated CpG DNA *in vitro* similar to the canonical CXXC domain (118). Genomic distribution of IDAX as determined by Chromatin IP (ChIP) showed that about 40% of IDAX peaks were enriched in the promoter/TSS, which suggested that IDAX acted as a cofactor to recruit *TET2* to target sites. Unexpectedly, however, overexpression of IDAX actually reduced the global level of 5-hmC (118), despite finding no changes in the *TET2* mRNA levels. Additionally, while the variant CXXC domain of IDAX was able to directly associate with the catalytic domain of *TET2*, IDAX does not block TET2 enzymatic activity directly. Instead, it appears that IDAX destabilizes the TET2 protein, which is then degraded through caspase 3 and 8 (118).

An important regulatory control on TET activity has been recently discovered with the finding of *Isocitrate Dehydrogenase-1* and *-2* (*IDH1/2*) mutations in a variety of tumors, including AML and MDS. IDH1/2 are enzymes that play an important role in the tricarboxylic acid cycle (TCA). Heterozygous, gain of function mutations in these enzymes have been found in high frequency in myeloid malignancies (119, 120). These mutations cause *IDH1/2* to produce an oncometabolite, 2-hydroxyglutarate (2-HG), instead of 2-OG (121). This is a competitive antagonist of 2-OGT enzymes, which in turn leads to severely reduced TET enzyme activity. As predicted, Figueroa et al. (122) found that *IDH1/2* mutations in AML patients lead to genome wide DNA hypermethylation signatures. While the population of patients with genetic *IDH1/2* mutations does not overlap with *TET2* mutations, these different mutations have essentially synonymous hypermethylation signatures. This suggests that *IDH1/2* and *TET2* mutations phenocopy one another and therefore do not confer additional selective advantages during clonal evolution in these diseases.

## *TET2* Mutations in AML and MDS

Loss of functional *TET2* has been extensively reported in both AML and MDS. In addition to translocation fusions with *MLL*, DNA FISH studies have shown that *TET2* is frequently deleted in both malignancies (123). *TET2* mutations have been identified in 12–24% of AML patients and 7–26% of MDS patients (66–68). Most mutations in *TET2* are heterozygous and the presence of mutations carries a poor prognosis in either malignancy (66). Missense mutations of *TET2* in AML and MDS patients are commonly located in the catalytic domain, spacer region, or the Cys-rich domain (Figure 2), or were nonsense or frameshift mutations. Notably, as many of these mutations can be found in flow cytometry defined HSC or early progenitor cells from patients with AML or MDS, *TET2* mutations are hypothesized to be possible pre-leukemic mutations (67, 68, 124).

A number of recent studies have focused on delineating the functional role TET2 plays in hematopoiesis. First, expression of *TET2* with mutations at its predicted Fe (II) and 2-OG binding residues led to decreased 5-hmC levels in cell lines compared to expression of wild-type enzyme, suggesting that the common mutations in these residues occurring in AML are loss of function. Loss of *TET2* has important phenotypic consequences in hematopoiesis. Transduction of *TET2* shRNA in bone marrow stem/progenitor cells impaired myelopoiesis (73), while both germline and conditional knockout of *Tet2* in mice in HSC leads to granulomonocytic (GM) lineage skewing at the expense of the erythroid and lymphoid lineages, as well as increased 5-mC level and decreased 5-hmC (125–127). Additionally, loss of *TET2* in human CD34^+^ cord blood recapitulates findings in mice, with differentiation skewing along GM lineages in *ex vivo* culturing conditions, along with increased HSPC self-renewal (128). To summarize, in both mouse and human models, *TET2* loss appears to promote GM lineage skewing and increases the self-renewal capacity of HSPC with aberrant ratios between 5-mC and 5-hmC.

The detailed mechanism of how *TET2* mutations propagate leukemic and pre-leukemic states in myeloid malignancies remains poorly understood. As expected, many studies reported that *TET2* mutations or depletion resulted in decreased 5-hmC globally (73, 125, 128). While the functional importance of DNA methylation at CpG islands has been correlated with transcription silencing, it appears that demethylation reactions catalyzed by TET2 might be more nuanced. Specifically, it was found by Ko et al. (73) that DNA hypermethylation profiles in bone marrow samples from patients harboring *TET2* mutations was enriched predominantly in non-CpG sites, while CpG islands were actually hypomethylated. Other groups have confirmed that the hypermethylation phenotype of *TET2* mutations appears to be principally outside of CpG islands. Yamazaki and colleagues (129) reported also did not detect changes in DNA methylation in CpG islands caused by *TET* mutations but instead detected hypermethylation at non-CpG islands. Rasmussen and colleagues recently reported that depletion of *Tet2* in pre-leukemic hematopoietic cells in mice had little impact on the methylation status of CpG islands and promoters but rather led to progressive DNA hypermethylation at enhancer elements (130). While the failure to detect differential DNA methylation at CpG islands in the presence of *TET2* mutations could be due to the degradation of mutant TET2 by IDAX as described above, this is at best speculative to date given the lack of direct evidence available. Finally, as the functional role of DNA methylation in non-CpG sites such as enhancers and gene bodies are largely unknown, how *TET2* or *IDH1/2* mutations lead to leukemia promoting transcriptional changes through hypermethylation in these sites is unclear; while TET2 was found to be significantly enriched with H3K4me1 and transcription factor p300 at the enhancer regions (131), whether TET2 is required for establishing these enhancers marks, whether mutant *TET2* changes the behavior of these cis regulatory regions, and how this ultimately perturbs transcriptional networks is still unexplored.

## Discussion and Perspectives

The high frequency of mutations in epigenetic regulators indicates that epigenetic deregulation may play a critical role in the pathogenesis of certain myeloid malignancies. The finding of these mutations both in malignant cells of AML and MDS as well as within the phenotypically normal HSC of patients indicates that these mutations may play a critical role in the pre-malignant phase of oncogenesis and evidence from single cell sequencing studies suggest that these cells can serve as reservoirs for disease relapse. In light of the increasing evidence for pre-LSC in both primary and relapse AML and in MDS, it is now critical to develop a more comprehensive understanding of what these cells are, how they are separated from other clones in CHIP, and how mutations in epigenetic regulators prime these pre-LSC toward oncogenesis. While a causal role for *TET2* and *DNMT3A* mutations is likely given that they are some of the most frequently mutated genes found in pre-LSC of AML and MDS patients, how exactly these mutations lead to leukemogenesis is still far from understood. For one, it is not clear how these mutations and their associated effects on global and local DNA methylation drive gene expression aberrations that impair normal hematopoiesis. Recent work has begun to shed some light on transcriptional and cell biological mechanisms that play a role in the formation of pre-LSC and their progression (132); however, it is unclear how exactly these transcriptional changes prime cells to become leukemic after acquisition of another genetic hit. The major limitation in answering these questions is technical: at present there exist no reliable cell surface markers that unambiguously separate pre-LSC from non-leukemic HSC clones. Therefore, deciphering the deregulated transcriptional programs occurring in pre-LSC, and how they relate to changes in DNA methylation cannot be readily achieved using ensemble approaches. Second, normal HSC are already documented as transcriptionally and functionally heterogeneous (133). As such, even single-cell gene expression technology like single-cell RNA-seq may only be adequate for identifying these transcriptional programs if single cell NGS or MeDIP-seq is performed concomitantly. At the time of writing, this technique has yet to be reported and is likely to represent an enormous technological challenge. Therefore, identifying what transcriptionally constitutes a truly “normal” versus “pre-leukemic” HSPC will be challenging given the present technology. Second, why certain mutations significantly enrich with *TET2* and *DNMT3A* is not well understood. One possibility is that loss of *TET2* or *DNMT3A* specifically contributes to increased mutation rates at these cooperating hits. Another possibility is that these hits are randomly generated but are selectively able to complement *TET2* or *DNMT3A* to drive leukemic evolution. As the clinical and biochemical implications of these two models differ significantly, establishing which contributes to AML and MDS is critical to developing a full understanding of these conditions and possibly developing novel therapeutics. In either case, however, the preponderance of these mutations in pre-LSC strongly suggests an important pathogenic role as leukemia initiation. Third, while the importance of DNA methylation in transcriptional regulation is well documented, a detailed mechanism of how DNA methylation patterns are established and maintained is far from complete. How these processes are locally augmented during normal hematopoietic differentiation is similarly unknown. The fact that multiple studies looking at loss of *TET2* or *DNMT3A* reported similar phenotypic changes in HSC (namely GM skewed cell fate, increased self-renewal capacity, and global changes of DNA methylation status) seems to indicate that aberrant methylation patterns have robust effects on hematopoietic differentiation. The fact that hypo- and hyper-methylation patterns can have similar phenotypic consequences indicates that perhaps the marks *per se* are not as important as the appropriate ratio of these marks across many local regions of the genome in the same cell. Further complicating matters is the finding that these methylation patterns do not appear to correlate well with gene expression changes in AML and MDS samples, while mutations in both *TET2* and *DNMT3A* clearly have prognostic implications and participate in leukemogenesis. Given these points, deciphering the language of these methylation patterns and determining how they dictate hematopoietic differentiation is a major focus of current research.

A substantial amount of research will be still required to fully understand how these epigenetic factors behave in both normal and malignant hematopoiesis. With technological advancements, particularly in NGS and single cell techniques, many of the counterintuitive observations made regarding these enzymes may be elucidated. Given the inherent heterogeneity of normal HSPCs, it is quite likely that single cell transcriptomics and epigenomics may be ultimately required to fully understand how and when these factors become relevant in promoting LSC transformation. Despite the current technical limitations, however, the discovery of these mutations in pre-LSC has blossomed exciting new lines of research in both AML and MDS, diseases with classically poor prognoses and little therapeutic advances over the past few decades. Although it appears that the role epigenetic regulators play in leukemia initiation will be complex, those functions are likely to fundamentally alter current paradigms about how these myeloid malignancies develop and therefore may offer novel management avenues in the future.

## Author Contributions

All authors listed, have made substantial, direct, and intellectual contribution to the work and approved it for publication.

## Conflict of Interest Statement

The authors declare that the research was conducted in the absence of any commercial or financial relationships that could be construed as a potential conflict of interest.
